# Psychopathology and Quality of Life in patient residing in Specialised Residential Units compared to those residing in the Community in the Sligo Leitrim Mental Health Service

**DOI:** 10.1192/j.eurpsy.2026.11647

**Published:** 2026-07-31

**Authors:** C. Yilmaz, D. Adamis, E. McGuire

**Affiliations:** 1 Psychiatry, Sligo Leitrim Mental Health Services, Sligo; 2 Psychiatry, National University of Ireland Galway, Galway, Ireland

## Abstract

**Introduction:**

Since the mid-20th century, mental health reform has emphasized deinstitutionalisation, leading to supported accommodation models. In Ireland, *Planning for the Future* (1984) and *A Vision for Change* (2006) promoted independent living but acknowledged that some patients require long-term care in Specialised Residential Units (SRUs). Although SRUs aim to be non-institutional, concerns of “trans-institutionalisation” persist, with limited rehabilitation opportunities. Evidence suggests many patients can transition successfully to community living, with improved quality of life. While international studies have examined psychosocial outcomes across housing settings, Irish research has largely focused on former long-stay patients, with no direct comparisons between accommodation types.

**Objectives:**

Our primary objective was to assess psychopathology severity in SRU residents versus community patients, examining whether SRU patients experienced more positive or negative symptoms. Secondary objectives were to evaluate perceived quality of life and overall functioning in both cohorts.

**Methods:**

We carried out an observational cross sectional study of patient’s symptom profiles, their perceived quality of life and their overall level of functioning. The study was carried out in the 2 SRUs which are currently under the governance of the Rehab and Recovery Team, and in the community base, in Sligo. We collected data on symptoms using the PANSS. To assess QoL, we used the WHOQOL-BREF questionnaire. Global Assessment of Functioning (GAF) Scale was used to assess functionality, as well.

**Results:**

The study included 70 participants: 51 community-dwelling and 19 SRU residents. Both groups were mostly male, single, and unemployed, with SRU residents significantly older (χ²=7.91, p=0.005). PANSS and GAF scores showed no differences in symptoms or functioning (χ²=10.05, p=0.122). QoL ratings across all domains indicated moderate satisfaction, without variation. Social engagement differed (χ²=9.97, p=0.041): 78.9% of SRU residents had <10 hrs/week with significant others vs. 43.1% in the community, while 31.4% of community patients had >50 hrs (none in SRUs). Siblings were most cited in both, though SRU residents relied more on multiple contacts (55.6% vs. 18%) and community patients reported more diverse supports.

**Conclusions:**

This study found no significant differences in psychopathology, functioning, or QoL between SRU and community residents, challenging assumptions that higher symptom load necessitates higher support. Differences were most evident in age and social contact, with SRU residents experiencing greater isolation. These findings highlight the need to re-examine housing models, prioritise autonomy, and direct resources toward interventions that enhance social integration and quality of life.

**Disclosure of Interest:**

None Declared

